# Changes in postural balance associated with a woman's aging process

**DOI:** 10.1016/j.clinsp.2022.100041

**Published:** 2022-05-11

**Authors:** Guilherme Carlos Brech, Tatiana Godoy Bobbio, Kelem de Negreiros Cabral, Patrícia Mota Coutinho, Leila Regina de Castro, Luis Mochizuki, Jose Maria Soares-Junior, Edmund Chada Baracat, Luiz Eugênio Garcez Leme, Julia Maria D'Andréa Greve, Angélica Castilho Alonso

**Affiliations:** a Graduate Program in Aging Sciences, Universidade São Judas Tadeu (USJT), São Paulo, SP, Brazil; b Laboratory Study of Movement, Instituto de Ortopedia e Traumatologia do Hospital das Clínicas (IOT-HC) da Faculdade de Medicina da Universidade de São Paulo (FMUSP), São Paulo, SP, Brazil; c Universidade de Campinas (UNICAMP), Campinas, SP, Brazil; d University of St. Augustine for Health Sciences, Miami Campus, United States of America; e Disciplina de Ginecologia, Departamento de Obstetrícia e Ginecologia, Hospital das Clínicas, Faculdade de Medicina da Universidade de São Paulo (FMUSP), São Paulo, SP, Brazil

**Keywords:** Accidental falls, Aging, Postural balance, Stabilometric parameters

## Abstract

**Context:**

Aging causes a progressive worsening in postural balance, affecting functional independence and increasing the risk of falls.

**Objective:**

The aim of the study was to evaluate the effect of aging on the static balance in women from 50-years to 89-years of age.

**Design:**

This was a cross-sectional study, with 400 irregularly active women were evaluated and grouped by age: Group 6^th^ decade (age 50 to 59) ‒ 58 participants; Group 7^th^ decade (age 60 to 69) ‒ 214 participants; Group 8^th^ decade (age 70 to 79) ‒ 92 participants; Group 9^th^ decade (age 80 to 89) ‒ 36 participants. Postural balance was evaluated using a portable force platform in a standard standing position, with Eyes Open (EO) and Eyes Closed (EC).

**Results:**

In the two measurement conditions, the elderly women in Group 9^th^ decade presented mediolateral displacement and range, and mean velocity greater than the women's values in Groups 6^th^ and 7^th^ decade. In the EO e EC situation, the displacement was higher in the elderly Group 9^th^ decade compared to younger groups. Group 8^th^ has a mean velocity greater than Group 6^th^ decade in the EO situation.

**Conclusions:**

Posturography showed a decline in postural balance with advancing age, suggesting that the 9^th^ decade of life is a borderline age to this detriment due to an increase in postural instability.

## Highlights

• Aging process increases postural oscillations, worsening the postural balance.
• Poor postural balance is more evident in the elderly over 80-years old.
• Deterioration of the postural control systems and the increased risk of falls.
• Important identify factors related to falls and postural balance.

## Introduction

Aging does not necessarily mean disability; however, it increases the risk of injury, especially those resulting from falls. Falling is defined as an unexpected and abrupt event, which is often due to the loss of balance and the postural control failure mechanisms.^1^

Cox et al.^2^ report that 9.7% of emergency hospitals visits are due to falls. Falls in the elderly accounted for 32.6% of domestic accidents, and they are associated with a deficiency of postural balance.^2^ Falls are the common cause of major disability in the elderly. Secondary consequences include traumatic brain injuries, fractures, and chronic disease as strokes.^3^

Women are more vulnerable to falls because they have a decline in bone mass is 0.5% per year after the age of 40, and is more accentuated, around 3% per year, after menopause, caused by the end of estrogen production by the ovaries, combined with muscle weakness and decreased amplitude of movement in lower limbs, capable of causing stumbling and loss of postural balance. Insufficiency or deficiency of vitamin D can be associated with muscle hypotrophy and may increase the risk of falls.^4^

Postural balance is the capacity of maintaining the center of gravity within the base of support, which is important for the efficient and safe execution of the Activities of the Daily Living (ADL).^5^^,^^6^ The preservation of balance contributes to the functional independence of the elderly people and to prevention of falls helping to maintain the functional capacity.^7^^,^^8^

Age-related declines influence postural control mechanisms related to stability and function. Stability is related to physiological and biomechanical changes such as sarcopenia, resulting in reduced strength, loss of flexibility, mobility and, consequently, worsening of postural balance. On the other hand, the decline in function related to age and body morphology changes necessitate the use of different muscular combinations in support of postural goals; these self-organizing coordinative synergies provide optimized combinations of muscle activation to help maintain postural balance during the life.^9^

Furthermore, visual impairments and decline in somatosensory integration exacerbate postural stability,^8^ and the postural balance^8^ is aggravated by worsening of the visual and somatosensory system integration, which can increase the risk of falls in the elderly.^8^^,^^10^^,^^11^

Postural control in the human body is controlled by neuromuscular responses, which are essential in maintaining postural balance. These responses come from the nervous, sensorial, and motor systems.^12^ The adaptive responses in the elderly are slower, which causes greater postural oscillations and, consequently, a greater number of falls, given to these intrinsic factors.^5^ There is a decreased ability to adapt to an external stimulus, which leads to progressive loss of physical performance and greater vulnerability.^13^, ^14^, ^15^

Posturography (static postural balance evaluation) is being used as a tool to quantify this decline throughout life, and it can be helpful in the creation and implementation of fall prevention programs.^7^^,^^12^^,^^16^^,^^17^

Adaptive mechanisms become less efficient over the years, and in this study it was verified that the aging process increases the postural oscillations, with the consequent worsening of postural balance.

The aim of the study was to evaluate the effect of aging on the static balance in women from 50-years to 89-years of age.

## Methods

### Study location and ethical issues

This was a cross-sectional study performed at the Motion Study Laboratory of the Institute of Orthopedics and Traumatology, Hospital das Clinics, Faculty of Medicine, University of São Paulo. All participants gave their written informed consent to participate in this study, which was approved by the Ethics Committee at the Faculty of Medicine, University of São Paulo (registration number 320/09).

### Participants

This is a convenience sample size of 400 women. The inclusion criteria for this study were: a) Women aged 50 and over, b) Participant report of absence of vestibular, proprioceptive, auditory, or neurological impairment, and/or any mental disturbances or disorders; c) Participant report of no use of medications that might compromise postural balance; d) Participant report of absence of lesions, surgery, or disease that might have caused lower-limb joint limitations over the previous six months; e) Assessed absence of lower-limb dysmetria; and f) Assessed clinically normal gait, without claudication; g) Assessed by a validated quiz of physical activity, only include the participants with irregularly active: women who engage in physical activities less than 150 minutes per week; Participants were excluded if there was a presence of pain or inability to complete any of the tests.

### Assessments

Older adults were evaluated and screened in the Motion Study Laboratory of the Institute of Orthopedics and Traumatology, Hospital das Clinics, University of São Paulo School of Medicine.

All the research participants answered a questionnaire with demographic and anthropometric data prior to the assessment.

The postural balance assessment (posturography) was performed on a portable force platform (AccuSway Plus, AMTI®, MA, USA). For data acquisition, the force platform was connected to a signal-amplifying interface box (AMTI Accus gait PJB 101, Watertown, MA) that was linked to a computer by means of an RS-232 cable. The data were gathered and stored using Balance Clinic® software, configured to a frequency of 100 Hz with a fourth-order Butterworth filter and a cutoff frequency of 10 Hz. All subjects underwent the test with standardized positioning in relation to the maximum width of the support base (smaller than hip-width), with arms along the body and the head looking straight at a target. The base of support was drawn on a paper on a fixed position on the force platform, corresponding to the anatomical points of the distal hallux phalanx, fifth metatarsal head, lateral and medial malleolus for each foot. Three measurements were made with Eyes Open (EO) and three with Eyes Closed (EC) for 60 seconds each, without any randomization. The arithmetic mean of the results was calculated from the three tests conducted under each condition and was processed using Balance Clinic® software. The parameters used to measure the subject's stability with eyes open and closed were the root mean square of the displacement from the COP in the medio-lateral and antero-posterior directions; maximum range displacement of the COP (sum of the maximum and minimum displacement/amplitude) antero-posterior and medio-lateral directions; mean velocity calculated from the total displacement of the COP in all directions; area of 95% from an ellipse of the total COP displacement.^18^^,^^19^

### Statistical analysis

Data were analyzed in the GraphPad PRISM software (GraphPad Software, Inc.) presented by mean and standard deviation. The data distribution was evaluated by the Kolmogorov Smirnov test, followed by the single-factor ANOVA test with Bonferroni Post Hoc. The significance level adopted was p≤0.05 for all statistical analyses.

## Results

The 400 women were grouped by age: Group 6^th^ decade (age 50 to 59) ‒ 58 participants; Group 7^th^ decade (age 60 to 69) ‒ 214 participants; Group 8^th^ decade (age 70 to 79) ‒ 92 participants; Group 9^th^ decade (age 80 to 89) ‒ 36 participants.

There was no difference in anthropometric characteristics (body weight, height, and body mass index) between the groups (Table 1).

**Table 1:** Anthropometrics characteristics by age groups (decade).

	Group 6^th^	Group 7^th^	Group 8^th^	Group 9^th^
	50‒59 years	60‒69 years	70‒79 years	80‒89 years
	(n = 58)	(n = 214)	(n = 92)	(n = 36)
	Mean (SD)	Mean (SD)	Mean (SD)	Mean (SD)
**Age (years)**	56.8 (±2.3)	63.8 (±2.7)	73.9 (±2.5)	82.6 (±2.2)
**Body weight (kg)**	64.2 (±10.3)	69.1 (±14.0)	69.1 (±13.0)	65.4 (±16.0)
**Height (m)**	154 (±0.0)	155 (±0.0)	155 (±0.0)	154 (±0.0)
**BMI (kg/m^2^)**	27.0 (±4.2)	28.3 (±4.9)	28.5 (±4.5)	27.0 (±5.4)

Anova test and Bonferroni pos hoc.

p-value ≤ 0.05.

All posturography variables are related to the displacement of the Center of Pressure (COP) in the antero-posterior and medio-lateral directions. The values of the postural instability assessment using the force platform are shown in Table 2.

**Table 2:** Static balance with eyes open and closed by age group (decade).

	Group 6^th^	Group 7^th^	Group 8^th^	Group 9^th^
	50‒59 years	60‒69 years	70‒79 years	80‒89 years
	(n = 58)	(n = 214)	(n = 92)	(n = 36)
	Mean (SD)	Mean (SD)	Mean (SD)	Mean (SD)
**Eyes open**				
Medio-lateral displacement (cm)	0.24 (±0.10)	0.26 (±0.12)	0.29 (±0.15)	0.33 (±0.11)
Anteroposterior displacement (cm)	0.41 (±0.12)	0.43 (±0.18)	0.44 (±0.18)	0.46 (±0.15)
Range medio-lateral (cm)	1.24 (±0.37)	1.39 (±0.71)	1.55 (±0.81)	1.83 (±0.69)
Antero-posterior range (cm)	2.09 (±0.51)	2.29 (±0.94)	2.40 (±1.03)	2.61 (±0.80)
Mean velocity (cm/s)	0.87 (±0.28)	0.94 (±0.30)	1.06 (±0.61)	1.22 (±0.55)
Area 95% (cm²)	1.89 (±1.11)	2.06 (±1.47)	2.41 (±1.77)	2.90 (±2.00)
**Eyes closed**				
Medio-lateral displacement (cm)	0.25 (±0.99)	0.26 (±0.12)	0.28 (±0.15)	0.35 (±0.14)
Anteroposterior displacement (cm)	0.44 (±0.14)	0.48 (±0.26)	0.46 (±0.18)	0.49 (±0.18)
Medio-lateral range (cm)	1.39 (±0.64)	1.53 (±0.82)	1.66 (±1.10)	1.86 (±0.81)
Range anteroposterior (cm)	2.48 (±0.77)	2.85 (±1.03)	2.82 (±1.10)	2.80 (±1.10)
Mean velocity (cm/s)	1.04 (±0.34)	1.19 (±0.43)	1.27 (±0.59)	1.44 (±0.62)
Area 95% (cm²)	2.16 (±1.62)	2.42 (±1.69)	2.37 (±1.76)	3.10 (±2.17)

### Eyes open condition

The medio-lateral displacement of the COP of Group 9^th^ decade was higher compared to Groups 6^th^ (p = 0.009) and 7^th^ (p = 0.01) decade. The medio-lateral range of the COP of Group 9^th^ decade was higher compared to Groups 6^th^ (p = 0.001) and 7^th^ (p = 0.003). The Group 8^th^ decade was higher compared to Group 6^th^ (p = 0.05) decade.

The antero-posterior range displacement of the COP of Group 9^th^ decade was higher when compared to Groups 6^th^ decade (p = 0.04).

Regarding the displacement velocity of the COP, Group 9^th^ decade showed higher values compared to Groups 6^th^ (p = 0.001) and 7^th^ (p = 0.002). Also, a significant difference in displacement velocity of the COP was also founded between Group 8^th^ and Group 6^th^ decade (p = 0.05).

**Fig. 1:**
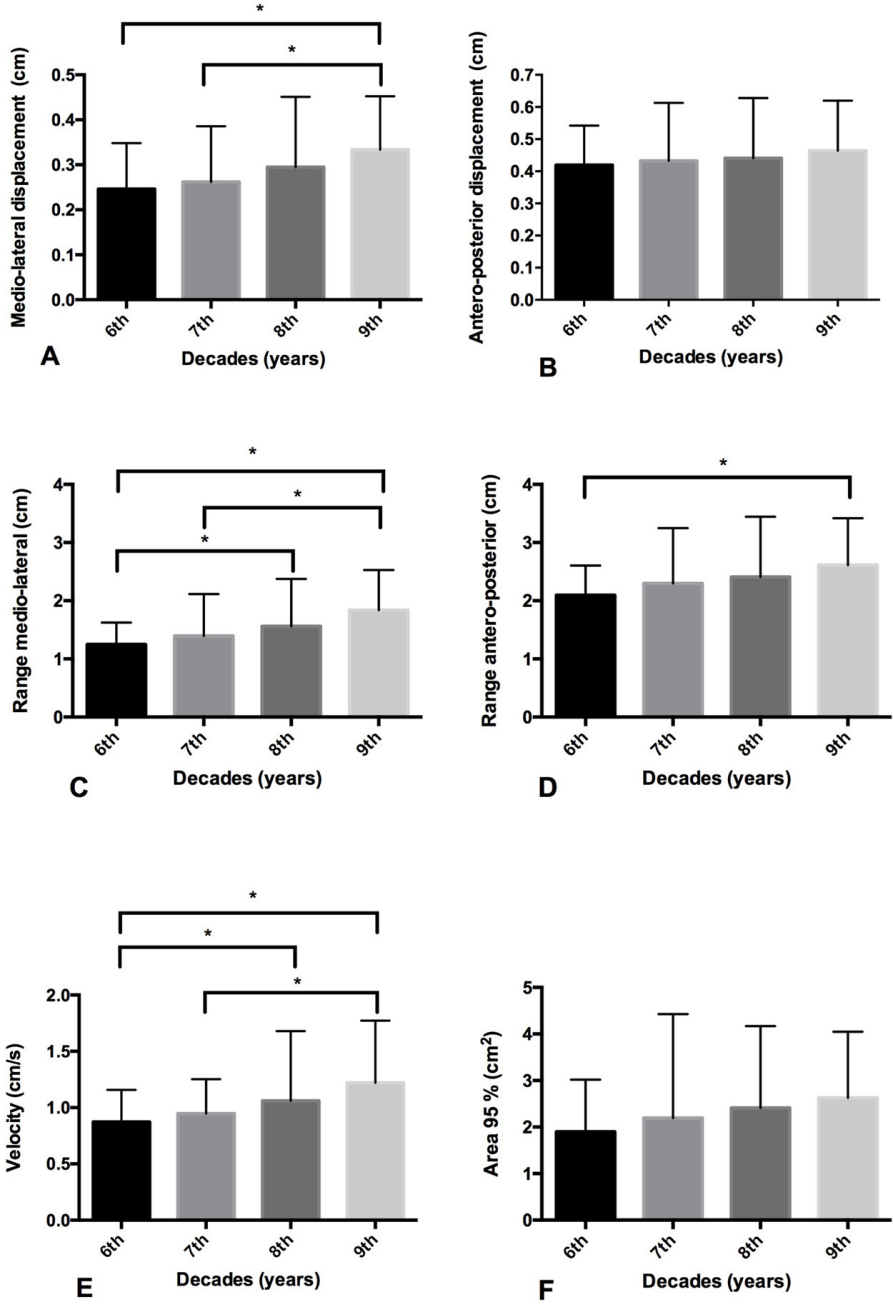
Comparison of postural balance with eyes open between age groups. (A) Latero-lateral COP displacement; (B) Antero-posterior COP displacement; (C) Mean velocity calculated from the total displacement of the COP in all directions; (D) Range: sum of the maximum and minimum displacement of the COP in latero-lateral direction; (E) Range: sum of the maximum and minimum displacement of the COP in anteroposterior direction; (F) Area of 95% from an ellipse of the total COP displacement. * p-value ≤0.05 ANOVA with Bonferroni Correction (Pos hoc).

There was no difference between age groups in a mean of 95% area of the ellipse displacement of COP and anteroposterior displacement of the COP (Fig. 1).

### Eyes closed condition

The medio-lateral displacement of the COP in Group 9^th^ decade was higher compared to 6^th^ (p = 0.003) and 7^th^ (p = 0.006) decades groups. Also, in the range, medio-lateral displacement of the COP of Group 9^th^ decade was higher when compared to 6^th^ (p = 0.030) decades groups.

**Fig. 2:**
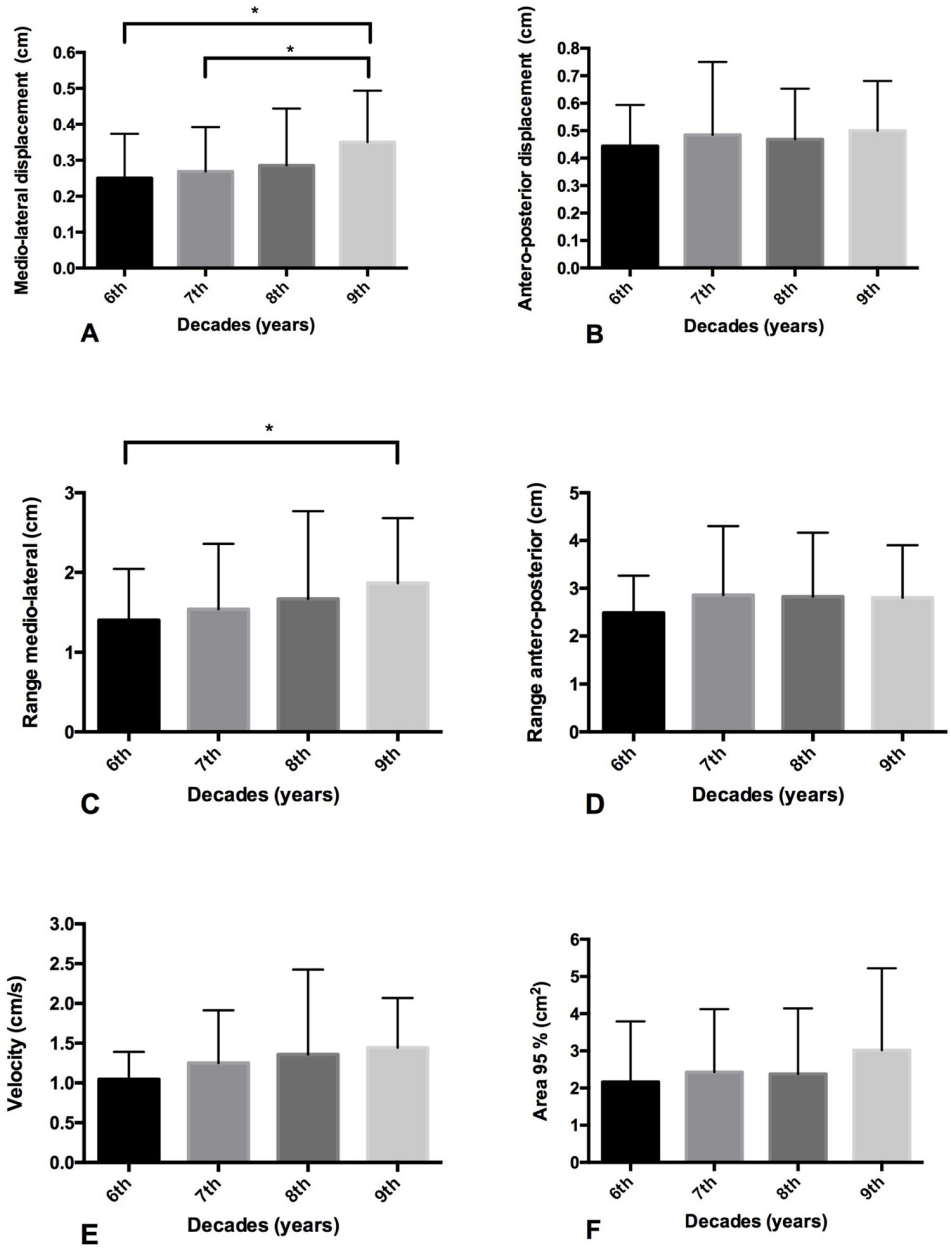
Postural balance comparison between age group with eyes closed. (A) Latero-lateral COP displacement; (B) Anteroposterior COP displacement; (C) Mean velocity calculated from the total displacement of the COP in all directions; (D) Range: sum of the maximum and minimum displacement of the COP in latero-lateral direction; (E) Range: sum of the maximum and minimum displacement of the COP in anteroposterior direction; (F) Area of 95% from an ellipse of the total COP displacement. * p-value ≤0.05 ANOVA with Bonferroni Correction (Pos-hoc).

There was no difference between age groups in antero-posterior displacement and range, medio-lateral range of the COP, displacement velocity of the COP, and a mean of 95% area of the ellipse displacement of COP. Group 8^th^ decades group did not present any difference between the other decades groups in all postural balance variables, seen in Fig. 2.

## Discussion

In this study, it was verified that the aging process increases the postural oscillations, with the consequent worsening of postural balance. Variances between the groups are most evident in the elderly over 80-years old due to greater deterioration of the postural control systems and the increased risk of falls. Hartholt et al.^20^ reports an increase in the number of deaths of Dutch people aged 80 and over due to falls: 391 in 2000 and 2.501 in 2016. The overall mortality rate per 100.000 population increased from 78.1 (95% CI 70.4‒85.9) in 2000 to 334.0 (95% CI 320.9‒347.1) in 2016. The increase in the elderly population aged 80-years and over shows that identifying the factors related to falls should be taken into account in setting priorities, and the decline of postural balance may be the most important factor.

Significant postural balance losses were seen from the 9^th^ decade of life (when compared to 6^th^ and 7^th^ decades’ groups. The deterioration was observed in both evaluation conditions – eyes open and closed, and in all variables. Worsening of the postural balance is progressive and slow because the humans adapt themselves to the alterations of the senescence and try to maintain the correct postural responses to the physical and environmental demands. However, from the 9^th^ decade, the organism can no longer maintain and activate the compensatory mechanisms, and the deficiencies are more evident, making it an aggravating circumstance of domestic falls, which are usually associated with changing positions, and engaging in tasks of daily living. Garcia et al.,^21^ in 2006 reported a 50% increase in falls among the elderly with 80-years or more when compared to the age group of 65 to 79 years. No studies showed the decline of postural balance comparing decades, however, some studies suggest a strong association between age and postural balance decrease.^22^, ^23^, ^24^

With eyes open, many variables of displacement and velocity were greater in 9^th−^decade group compared to younger groups (6^th^ and 7^th^ decades groups). With eyes closed, only medio-lateral displacement and range were greater in 9^th^ decade group compared to 6^th^ and 7^th^ decades, which were the youngest groups. Possibly, the differences seen in the evaluations with eyes closed occurred due to the fact that this condition was more challenging, by withdrawing visual control and medio-lateral sway is predictive of falls among elderly people.^25^ Occlusion of vision may make possible deficiencies in other balance control systems more evident, such as vestibular and sensorimotor systems. It was observed that the mean and standard deviation were higher in all groups in the EC evaluation when compared to EO.

Another possible explanation for the antero-posterior postural balance preservation are strategies used by the body to maintain stability. The control of antero-posterior balance is controlled by muscular responses that occur from distal to proximal strategies and initiate from the ankles to the trunk.^18^ Strategies for maintaining medio-lateral stability occur mainly at the hip and trunk, and less occurs at the ankle joint.^18^ The main medio-lateral movement of the body is a lateral movement from the pelvis with adduction of one leg and abduction of the other one. The movement from the ankle is only used in narrow places, which prevents the hip from moving. In the medio-lateral slope, the load displaced to one side is also assumed by the other. The hip abductors (gluteus medius and tensor fascia latae) and hip adductors are activated in both lower extremities, well before the ankle muscles. When assessing subjects with eyes open, Group 9^th^ decade showed greater oscillation in many the measured variables. With the occlusion of vision, the oscillations increased, but the differences between 9^th^ decade group in medio-lateral discpalecement and the range to 6^th^ and 7^th^ decade groups remained the same. Tomomitsu et al.,^26^ reported that with the occlusion of vision, there is a greater demand for the adjustment of the sensorimotor system in order to maintain the balance, even in stable semi-static and non-challenging situations.

Melzer et al.^27^ have shown that increased COP displacement in the medio-lateral plane is associated with recurrent falls. The eyes closed condition seems to be considered the best predictor for recurrent falls.^28^^,^^29^

The multifactorial aspects of balance and the use of static posturography appear to be the limiting factors in this study. However, the posturography has been shown to be a good tool to identify decline in balance as people age, data were consistent with Fujita et al.^22^

Balance maintenance is an acquired motor skill that improves with fitness throughout life. Maintenance of postural control can be learned by individuals, however, the adaptive mechanisms become less efficient over the years, particularly after the age of 80. The projected increase of population aging is accelerated, and it will bring many elderly people over 80-years. Fall prevention programs with strategies to improve postural stability and balance will be essential to maintain the functional capacity and quality of life of that population.

Posturography showed a decline in postural balance with advancing age, suggesting that the 9^th^ decade of life is a borderline age to this detriment due to an increase in postural instability.

## Authors’ contributions

Guilherme Carlos Brech: Investigation and Writing-original draft and Supporting Investigation and Writing-review & editing.

Tatiana Godoy Bobbio: Supporting Investigation and Writing-review & editing.

Kelem de Negreiros Cabral: Supporting Investigation and Writing-review & editing.

Patrícia Mota Coutinho: Supporting Investigation and Writing-review & editing.

Leila Regina de Castro: Supporting Investigation and Writing-review & editing.

Luis Mochizuki: Formal analysis and Writing-review & editing.

Jose Maria Soares-Junior: Supporting Investigation and Writing-review & editing.

Edmund Chada Baracat: Supporting Investigation and Writing-review & editing.

Luiz Eugênio Garcez Leme: Supervisor and Writing-review & editing.

Julia Maria D'Andréa Greve: Supervisor and Writing-review & editing.

Angélica Castilho Alonso: Formal analysis and Writing-review & editing.

## Conflicts of interest

The authors declare no conflicts of interest.
